# Brain Microglial Activation in Chronic Pain-Associated Affective Disorder

**DOI:** 10.3389/fnins.2019.00213

**Published:** 2019-03-15

**Authors:** Ellane Eda Barcelon, Woo-Hyun Cho, Sang Beom Jun, Sung Joong Lee

**Affiliations:** ^1^Department of Neuroscience and Physiology and Dental Research Institute, School of Dentistry, Seoul National University, Seoul, South Korea; ^2^Department of Brain & Cognitive Sciences, Ewha Womans University, Seoul, South Korea

**Keywords:** brain microglia, microglial activation, chronic pain, depression, TNF-α

## Abstract

A growing body of evidence from both clinical and animal studies indicates that chronic neuropathic pain is associated with comorbid affective disorders. Spinal cord microglial activation is involved in nerve injury-induced pain hypersensitivity characterizing neuropathic pain. However, there is a lack of thorough assessments of microglial activation in the brain after nerve injury. In the present study, we characterized microglial activation in brain sub-regions of CX3CR1^GFP/+^ mice after chronic constriction injury (CCI) of the sciatic nerve, including observations at delayed time points when affective brain dysfunctions such as depressive-like behaviors typically develop. Mice manifested chronic mechanical hypersensitivity immediately after CCI and developed depressive-like behaviors 8 weeks post-injury. Concurrently, significant increases of soma size and microglial cell number were observed in the medial prefrontal cortex (mPFC), hippocampus, and amygdala 8 weeks post-injury. Transcripts of CD11b, and TNF-α, genes associated with microglial activation or depressive-like behaviors, are correspondingly upregulated in these brain areas. Our results demonstrate that microglia are activated in specific brain sub-regions after CCI at delayed time points and imply that brain microglial activation plays a role in chronic pain-associated affective disorders.

## Introduction

Neuropathic pain is a form of pathological chronic pain caused by injury or dysfunction of the nervous system that affects tens of millions of people worldwide (Goldberg and McGee, 2011; Raffaeli and Arnaudo, 2017). For several decades, in-depth and comprehensive studies have addressed the pathology of neuropathic pain, but the pathogenic mechanisms remain elusive and efficient treatments for this devastating disease are limited. Accumulating evidence indicates that spinal cord microglia play a critical role in the development of neuropathic pain (Tsuda et al., 2004; Coull et al., 2005; Beggs and Salter, 2010; Zhuo et al., 2011). According to this pathogenic model, injured nerve-derived signals induce activation of the spinal cord microglia and subsequent pain-related gene expression. This in turn sensitizes pain-transmitting neurons or neural circuits, resulting in central pain sensitization at the spinal cord level and neuropathic pain (Beggs et al., 2012; Ferrini and Koninck, 2013; Tsuda et al., 2013).

Chronic pain patients, including those suffering from neuropathic pain, often experience comorbid affective disorders. For instance, more than half of neuropathic pain patients report depression and cognitive deficits (Povedano et al., 2007; Liu et al., 2017). Likewise, chronic neuropathic pain induced by peripheral nerve injury in animal models is accompanied by depressive-like behavior and memory loss in behavioral studies (Dimitrov et al., 2014; Gui et al., 2016). These findings indicate that not only the sensory circuitry in the spinal cord but also diverse supra-spinal regions involved in affective brain functions are affected by chronic pain conditions including neuropathic pain. Of note, recent studies implicate aberrant microglial activation in these affective brain dysfunctions. For instance, increased microglial activation was detected post-mortem in the prefrontal cortex, anterior cingulate cortex, and hippocampus of major depression disorder (MDD) patients (Steiner et al., 2008; Haarman et al., 2014; Torres-Platas et al., 2014). Furthermore, impaired microglia function was implicated in neuropsychiatric disorders (Blank and Prinz, 2013). These prior documents suggested that brain microglial activation due to peripheral nerve injury is involved in the development of these chronic pain-associated affective disorders. Thus far, studies of brain microglial activation in the context of nerve injury-induced neuropathic pain provided conflicting results. While several studies show microglial activation in brain regions including the hippocampus, prefrontal cortex, amygdala, and nucleus accumbens (Gui et al., 2016; Taylor et al., 2017; Xu et al., 2017) another study reported no obvious microglial activation in terms of cell proliferation and morphological changes in nerve-injured mouse brain (Zhang et al., 2008). Such discrepancies might be attributed to differences in methods of measuring microglial activation (microglia morphology vs. activation-related gene expression) or nerve injury models (nerve transection vs. constriction injury). Therefore, the effects of nerve injury on brain microglial activation remain to be resolved. Furthermore, most studies have characterized brain microglial activation within 2 weeks after peripheral nerve injury. Considering that the onset of chronic pain-associated affective disorders is relatively delayed, usually manifesting more than 1 month after injury (Dimitrov et al., 2014; Sieberg et al., 2018), microglial activation observed at early time points might not be relevant to chronic pain-associated affective disorders. Thus, in the present study we characterize brain microglial activation in various brain regions involved in affective components of pain at delayed time points in a chronic constriction injury (CCI)-induced neuropathic pain model utilizing CX3CR1^GFP/+^ mice, in which morphological microglia features can be assessed without immunohistochemistry.

## Materials and Methods

### Animals

All mice used in the study were male, housed four to five per cage and accommodated at a constant room temperature of 23 ± 2°C and a 12-hour light-dark cycle with access to food and water *ad libitum*. CX3CR1^GFP/+^ mice of C57BL/6 background were purchased from Jackson Laboratories (Bar Harbor, ME, United States) and C57BL/6 mice were obtained from DBL (Eumsung, Korea). Eight- to ten-week-old male CX3CR1^GFP/+^ mice were randomly divided into CCI and sham groups for the characterization of brain microglia (*n* = 5). Eight- to ten-week-old male C57BL/6 mice were used for behavior studies and grouped likewise. All surgical and experimental procedures were approved by the Institutional Animal Care and Use Committee at Seoul National University. Animal treatments were performed in accordance with the guidelines of the International Association of the Study of Pain.

### CCI Surgery

Mice were randomly assigned to undergo CCI or sham surgery. CCI was performed as previously described (Bennett and Xie, 1988). Briefly, animals were anesthetized under 1.5–2% isoflurane/oxygen. The right leg sciatic nerve was unilaterally exposed and loosely ligated with three 6-0 chromic gut sutures (Ailee Co., Busan, Korea) at about 1 mm distance from each other. The overlying skin and muscle were closed with the same silk sutures. Identical procedures were performed for the sham group except that the sciatic nerve was not ligated. Mice were returned to cages for the recovery period with free access to food and water after all surgical procedures.

### Behavior Tests

Each mouse was handled for 5 min every day for five days before conducting behavioral assessments. Mice were brought to the testing room 30 min prior to each behavioral test. Behavior tests including the open field test (OFT), tail suspension test (TST), and forced swim test (FST) were monitored using a computerized tracking system (SMART 3.0, Panlab Harvard Apparatus, Holliston, MA, United States).

### Von Frey Test

Mechanical allodynia was detected by assessing 50% withdrawal thresholds using a set of von Frey filaments (0.02–4 g, Stoelting, Wood Dale, IL, United States), following an up-down method, as previously described (Chaplan et al., 1994). Assessments were made before surgery or on weeks 1, 4, and 8 after surgery. Mice were placed in a cage with a wire mesh bottom allowing full access to the paws. The paws were touched with a series of von Frey filaments in ascending order of strength, at intervals allowing for resolution of behavioral response to the previous stimuli. Sharp paw withdrawal, paw licking, and flinching were interpreted as positive responses.

### Open Field Test

The OFT is a classic test of general locomotor activity in rodents and was performed in this study as previously described (Ramos et al., 2008; Fernando and Robbins, 2011; Haller and Alicki, 2012). Each mouse was placed in the center of an open arena (40 × 40 × 40 cm) and allowed to explore the arena for 5 min freely. The total distance traveled was analyzed to measure locomotor activity.

### Tail Suspension Test

To test depressive-like behavior, we conducted the TST as previously described (Steru et al., 1985). Briefly, mice were suspended using adhesive tape wrapped around tail 1 cm from the tip and tied to a hook in the observation chamber. Each mouse was suspended for 5 min and the duration of total immobility was measured.

### Forced Swim Test

The FST was performed as previously described (Fernando and Robbins, 2011). Mice were individually placed in glass cylinders (diameter and depth 10 × 25 cm) filled with water (25 ± 1°C) up to 19 cm. The mice were allowed to swim for 5 min and the duration of total immobility was measured. The water was changed after each trial.

### Immunohistochemistry

Mouse brains were perfused with 0.1 M PBS and then 4% PFA transcardially, post-fixed in the same solution, and transferred to 30% sucrose for 48 h. The brains were coronally cut into 40 μm-thick sections using a cryostat (CM3050S Cryostat, Leica, Wetzlar, Germany). Free-floating sections were put into cryoprotectant and stored at -80°C. Sections were washed three times with 0.1 M PBS containing 0.3% Triton X-100 (PBST) for 5 min each. After rinsing three times with 0.1 M PBS, sections were mounted on glass slides with mounting solution with DAPI (Vector Labs, Burlingame, CA, United States) and a cover slide. The sections were examined through an LSM 800 Confocal Laser Scanning Microscope (Carl Zeiss, Oberkochen, Germany). The GFP-labeled microglia were counted using ImageJ software (NIH). We analyzed brain areas from 3 to 4 sections per mouse from five mice for CCI and sham groups.

### Quantitative Real-Time PCR

cDNA was synthesized using total RNA isolated from frozen mouse brain areas investigated using Trizol (Thermo fisher Scientific, Waltham, MA, United States). qRT-PCR was performed using SYBR Green PCR Master Mix and an ABI Prism 7500 sequence detection system (Applied Biosystems, Foster City, CA, United States). The following primer sequences were used: GAPDH forward, 5′-AGG TCA TCC CAG AGC TGA ACG-3′; GAPDH reverse, 5′-CAC CCT GTT GCT GTA GCC GTA-3′; CD11b forward, 5′-ATG GAC GCT GAT GGC AAT ACC-3′; CD11b reverse, 5′-TCC CCA TTC ACG TCT CCC A-3′; TMEM119 forward, 5′-GGA TAG TGG ACT TCT TCC GCC A-3′; TMEM119 reverse, 5′-GGA AGG ACG ATG GGTA ATA GGC-3′; P2Y12 forward, 5′-TAA CCA TTG ACC GAT ACC TGA AGA-3′; P2Y12 reverse, 5′-ATC TTC GCA CCC AAA AGA TTG C-3′; P2RX7 forward, 5′-CTG GTT TTC GGC ACTG GA-3′; P2RX7 reverse, 5′-CCA AAGT AGG ACA GGG TGG A-3′TNF-α forward, 5′-TTG ACC TCA GCG CTG AGT TG-3′; TNF-α reverse, 5′-CCT GTA GCC CAC GTC GTA GC-3′; iNOS forward, 5′-GGC AAA CCC AAG GTC TAC GTT-3′; iNOS reverse, 5′-TCC ATC CAG TTG CCT TCT TGG-3′; IL-6 forward, 5′-CCA CGA TTT CCC AGA GAA CAT-3′ IL-6 reverse, 5′-TCC ATC CAG TTG CCT TCT TGG-3′. The mRNA level for each gene was normalized to the mRNA level of the GAPDH and presented as fold induction. Fold induction was calculated as previously described using the ^ΔΔ^CT method (Livak and Schmittgen, 2001).

### Statistics

All statistical tests were performed using IBM Statistics SPSS 23. Two-sample comparisons were conducted using Student’s *t* test, and multiple comparisons were analyzed using one-way or two-way ANOVA followed by *post hoc* tests to compare selected pairs of data. All data are presented as mean ± SEM. In all cases, *p* < 0.05 was considered statistically significant.

## Results

### CCI-Induced Mice Develop Depressive-Like Behaviors at a Delayed Time Point

**FIGURE 1:**
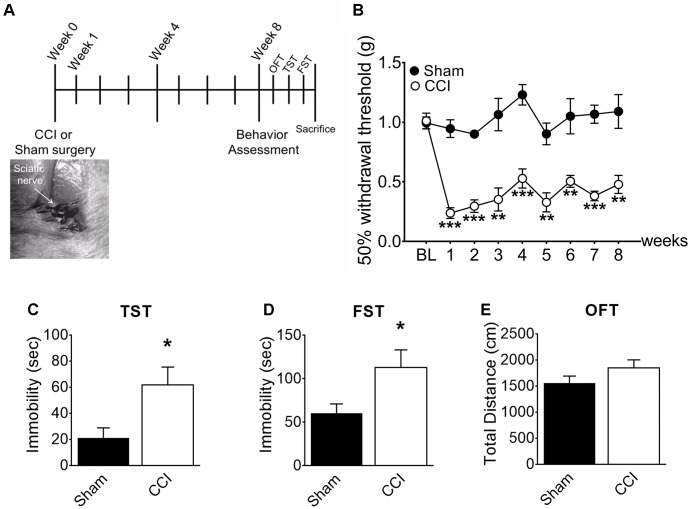
CCI-induced mice developed depressive-like behaviors. **(A)** Experimental timeline denoting weeks of behavior assessment. Bottom: CCI showing ligation of the sciatic nerve. **(B)** Mechanical allodynia tests were used to determine paw withdrawal thresholds before surgery (BL: baseline) and significant decreases of the paw withdrawal threshold 1, 4, and 8 weeks post-injury in CCI mice (*n* = 5, mean ± SEM, ^∗∗∗^*p* < 0.001, ^∗∗^*p* < 0.01). **(C)** TST and **(D)** FST showed significant increases of immobility time in the CCI group compared to the sham group (*n* = 5, mean ± SEM, ^∗^*p* < 0.05) indicating that depressive-like behaviors were induced 8 weeks post-injury. **(E)** Locomotor activity assessed in terms of total distance traveled in an open field test (OFT), showed no significant difference between groups (*n* = 5).

To induce chronic pain, we subjected mice to CCI of the right sciatic nerve (Figure 1A). After 1 week, the withdrawal threshold from mechanical stimuli measured by von Frey tests (Figure 1B) significantly decreased in the CCI-induced mice compared to sham-control mice (0.2g vs. 0.9 g), demonstrating induction of mechanical allodynia. CCI-induced mechanical allodynia persisted up to 8 weeks post-injury. To determine whether chronic pain accompanies affective brain disorders in a mouse neuropathic pain model, we monitored depressive-like behaviors in mice using the TST (Figure 1C) and FST (Figure 1D). Eight weeks after CCI injury, time spent immobile significantly increased in both TST and FST, indicating that mice developed depressive-like behavior at a delayed time point. Meanwhile, locomotor activity measured by total distance (Figure 1E) in OFT was not significantly altered in CCI-induced mice, indicating that the depressive-like behaviors observed are not due to defects in locomotor activity. These data indicate that CCI-induced mice develop depressive-like behaviors at a delayed time point (8 weeks).

### Microglia in the mPFC, Amygdala, and Hippocampus Are Activated at Delayed Time Points After CCI

**FIGURE 2:**
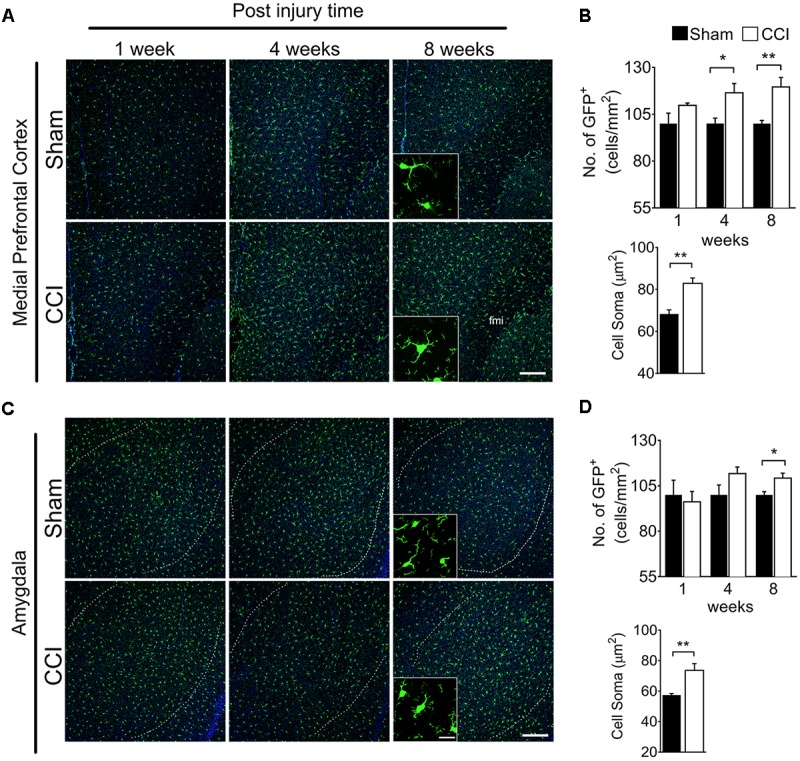
CCI-induced mice showed altered microglial morphology in the mPFC and amygdala at delayed time points. Representative images of **(A)** mPFC and a high magnification image at 8 weeks (inset) are shown. **(B)** Quantification of microglial cell number per square millimeter at 1, 4, and 8 weeks (*n* = 5, 3–4 tissue sections per animal, mean ± SEM, ^∗^*p* < 0.05, ^∗∗^*p* < 0.01) along with microglial soma area 8 weeks post-injury (*n* = 5, 3–4 tissue samples per section, 40 microglial cells, mean ± SEM, ^∗∗^*p* < 0.01) showing significant increases at 8 weeks in the CCI group compared to the sham group. Similarly, **(C)** representative images of the amygdala at 1, 4, and 8 weeks after injury, and a higher magnification image taken at 8 weeks (inset) are shown along with **(D)** microglial cell number and soma area quantification at 1, 4, and 8 weeks post-injury. Scale bar: 100 μm. Inset scale bar: 100 μm.

**FIGURE 3:**
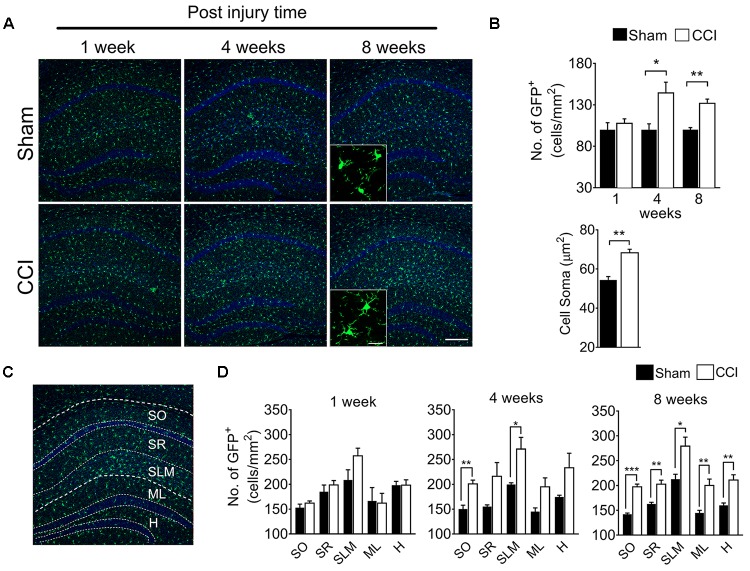
CCI-induced mice showed altered microglial morphology in hippocampal layers at delayed time points. **(A)** Representative images of the hippocampus and images taken at 1, 4, and 8 weeks post-injury under high magnification (inset) are shown. **(B)** Microglial cell number in the hippocampus shows significant increases at 4 and 8 weeks (*n* = 5, 3–4 tissue sections per animal, mean ± SEM, ^∗^*p* < 0.05, ^∗∗^*p* < 0.01) along with significant increases of the microglia soma area (*n* = 5, 3–4 tissue samples per section, 40 microglial cells, mean ± SEM, ^∗∗^*p* < 0.01). **(C)** Hippocampal layers, namely the stratum oriens (SO), stratum lacunosum moleculare (SLM), molecular layer (ML), and hilus (H) were demonstrated. **(D)** Hippocampal layer-specific microglial cell numbers were assessed at 1, 4, and 8 weeks post-injury and demonstrated significant differences in the CCI group compared to the sham group (*n* = 5, 3–4 tissue sections per animal, mean ± SEM, ^∗^*p* < 0.05, ^∗∗^*p* < 0.01, ^∗∗∗^*p* < 0.001). Scale bar: 100 μm. Inset scale bar: 100 μm.

To determine whether brain microglial activation is involved in chronic pain-associated depressive-like behavior exhibited by CCI-induced mice, we measured microglial activation in various brain sub-regions involved in affective brain functions including the medial prefrontal cortex (mPFC), amygdala, hippocampus, nucleus accumbens, periaqueductal gray, and habenula. For this purpose, we utilized CX3CR1^GFP/+^ mice in which morphological microglial activation can be directly assessed without immunostaining. Microglial activation is characterized by cell proliferation and cell body enlargement (Chen et al., 2012; Davis et al., 2017). Hence, at each time point (1, 4, and 8 weeks post-injury), GFP-positive microglial cell number and soma size were quantified. One week after CCI, microglial cell number and morphology were not significantly different in the mPFC (Figure 2A). However, at 4 weeks post-injury, we observed slight but significant increases in microglial cell number of 19.6%, and also a significant increase in soma size of 21.8%, typical of activated microglia (Figure 2B). The increases in microglial cell number and soma size lasted up to 8 weeks (Figure 2B). In the amygdala, we observed less increase in microglial cell number compared to mPFC, but upon quantification, the increase in cell number and soma area were also significant at 8 weeks post-injury (Figure 2C,D). Similar observations were noted in the hippocampus at 4 and 8 weeks, but not at 1 week after CCI (Figure 3A,B). When layer-specific differences were assessed, increased cell numbers were observed in the stratum oriens (SO), stratum lacunosum moleculare (SLM), and molecular layer (ML) at 4 and 8 weeks post-injury (Figure 3C,D). These data indicate that microglia in the mPFC, amygdala and hippocampus are activated at delayed time points after CCI.

### Microglia Are Not Generally Activated in the Whole Brain

**FIGURE 4:**
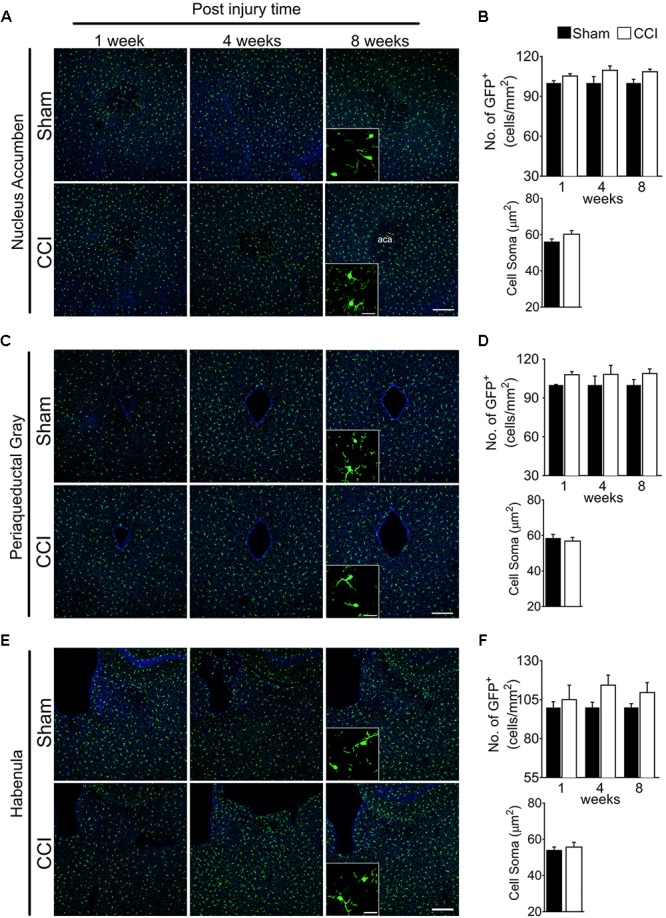
CCI did not show obvious changes of microglial morphology in the nucleus accumbens, periaqueductal gray, or habenula at any of the time points investigated. Representative images of the **(A)** nucleus accumbens, **(C)** periaqueductal gray, **(E)** habenula, and a high magnification image taken at 8 weeks post-injury (inset) are shown, along with **(B, D, F)** the areas of quantification of microglial cell number (*n* = 5, 3–4 tissue sections per animal, mean ± SEM) at 1, 4, and 8 weeks and soma area (*n* = 5, 3–4 tissue samples per section, 40 microglial cells, mean ± SEM) at 8 weeks post-injury showing no significant differences between the CCI group and sham group. Scale bar: 100 μm. Inset scale bar: 100 μm.

In the nucleus accumbens, periaqueductal gray, and habenula (Figure 4A,C,E), microglial cell numbers were not significantly increased in the CCI group compared to the sham group at any time points (Figure 4B,D,F). For the habenula, the medial and lateral habenula were quantified separately, but showed no significant differences between sham and CCI groups (data not shown). Likewise, no significant increases in microglial soma size were noted within the nucleus accumbens, periaqueductal gray, or habenula of CCI-induced mice compared to sham mice (Figure 4B,D,F). These data suggest that microglia are not generally activated in the whole brain, but in specific sub-regions involved in affective brain functions.

### CCI Differentially Induce Genes Related to Microglial Activation and Depressive-Like Behaviors

**FIGURE 5:**
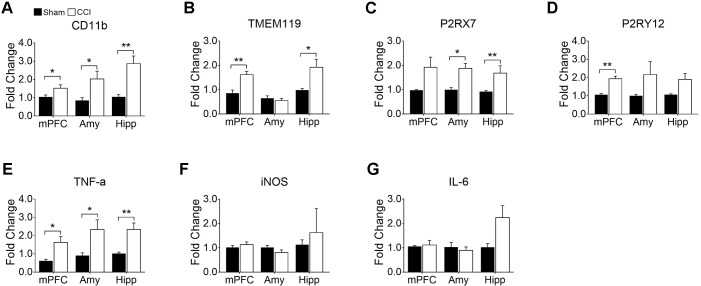
CCI-induced neuropathic pain induced changes in gene expression related to microglial activation and depression in the mPFC, amygdala (Amy), and hippocampus (Hipp) at delayed time points (8 weeks) post-injury. qRT-PCR showed significantly increased expression of **(A)** CD11b, **(B)** TMEM119, **(C)** P2RX7 **(D)** P2RY12, and **(E)** TNF-α in brain areas observed post-injury (*n* = 3∼6, mean ± SEM, ^∗^*p* < 0.05, ^∗∗^*p* < 0.01). However, qRT-PCR showed no significant differences in **(F)** iNOS and **(G)** IL-6 expression levels between CCI and sham groups post-injury (*n* = 3∼6).

Having identified microglial activation in the mPFC, hippocampus, and amygdala upon CCI, we further characterized microglial activation phenotypes in these brain regions by assessing gene expression profiles relevant to microglial activation and affective brain dysfunction. We found that CD11b mRNA expression was significantly increased in the mPFC, amygdala, and hippocampus, confirming microglial activation in these brain regions after CCI (Figure 5A). We further examined genes that are expressed by microglia in the central nervous system. TMEM119, a recently identified microglia-specific marker in both human and mouse (Bennett et al., 2016), was significantly increased in the mPFC and hippocampus but not in the amygdala (Figure 5B). P2RX7, a purinergic receptor reportedly increased in spinal microglia after peripheral nerve injury (Kobayashi et al., 2011), showed a significant increase in the amygdala and hippocampus, and a slight increase in the mPFC (*p* = 0.063) (Figure 5C). Whereas, P2RY12 (Gu et al., 2016) was significantly increased only in the mPFC but not in the amygdala (Figure 5D). Previous studies implicated aberrant hippocampal TNF-α expression in memory deficits (Ohgidani et al., 2016), and suggested that altered microglial M1/M2 polarization in the PFC is involved in depressive-like behavior (Xu et al., 2017). Therefore, we measured the mRNA expressions of these proinflammatory mediators. At 8 weeks post-injury, transcripts of TNF-α were increased more than 2-fold in the mPFC, amygdala, and hippocampus (Figure 5E). Meanwhile the transcript expressions of iNOS and IL-6 were not altered in the CCI group in any of the brain sub-regions that we examined (Figure 5F,G). These data demonstrate that CCI differentially induces genes related to microglial activation and depressive-like behaviors.

## Discussion

In this present study, we provide evidence that peripheral nerve injury induces brain microglial activation in the mPFC, amygdala, hippocampus at delayed time points, implicating microglial activation in chronic pain-associated depression. Studies of brain microglial activation in the context of chronic neuropathic pain are controversial. A previous study using CX3CR1^GFP/+^ mice concluded that the activation of microglia is selective to the spinal cord region, not to the brain, indicated by the observed lack of microglial activation in brain regions 1 week after peroneal nerve ligation (Zhang et al., 2008). A subsequent study showed that microglia are in fact activated in the habenula and ventral tegmental area at 2 weeks after CCI (Taylor et al., 2017). In that study, the authors suspected that the lack of observed microglial activation in a previous study might be due to the use of CX3CR1^GFP/+^ mice, in which a CXCR1 allele is replaced by GFP (Taylor et al., 2017). More recently, another study that also used the CX3CR1^GFP/+^ mouse line observed significant changes in microglial activation in the hippocampus 9 days after peripheral injury (Liu et al., 2017). Amid these differences, our investigation determined that, using CX3CR1^GFP/+^ mice, microglial activation is evident in the brain at delayed time points (more than 1 month) after the initiation of neuropathic pain, but changes are not observed at time points as early as 7 days post-injury. Therefore, our findings resolve the discrepancies between previous studies and suggest that nerve injuries induce brain microglial activation at relatively delayed time points, at least more than 1 week after injury. Alternatively, it is possible that brain microglial activation varies depending on the nerve injury model: that it is activated upon CCI injury, but not by common peroneal nerve ligation (Zhang et al., 2008; Ni et al., 2016; Liu et al., 2017).

Spinal cord microglia activation and its critical role in nerve injury-induced central pain sensitization has been well documented (Coull et al., 2005; Beggs and Salter, 2010; Beggs et al., 2012). According to these studies, microglia activation in the dorsal horn measured by morphological change peaks within 1 week after peripheral nerve injury. Furthermore, such morphological microglia activation accompanies drastic expression of proinflammatory genes such as TNF-α, IL-6, and iNOS (Lee et al., 2010; Kuboyama et al., 2011; Liu et al., 2017). Besides the temporal difference in activation after nerve injury, proinflammatory gene induction in the brain was relatively modest compared to that in the spinal cord; we detected gene induction of only TNF-α, but not IL-6 or iNOS. Therefore, it is likely that microglia activation mechanisms in the brain due to nerve injury are different from those in spinal cord microglia, which warrants future investigation.

We extended the scope of the present study to the development of depressive-like behaviors that may bridge observed changes in the brain microglia of our neuropathic pain animals with affective disorders. Previous studies showed that nerve injury induces alterations of microglial morphology in brain areas related to sensory functions (Descalzi et al., 2017; Taylor et al., 2017). In our study, we confirmed that depressive-like behaviors are also manifested at delayed time points, when microglia showed altered morphology. We investigated gene expression related to depressive-like behaviors in various brain areas, particularly the mPFC, amygdala and hippocampus. Recent studies have suggested that adaptive changes in the gene expression profiles in the brains of neuropathic pain-induced mice are similar to those of depression-induced mice (Descalzi et al., 2017), in which increased inflammatory cytokines are expressed (Xu et al., 2017). Among these pro-inflammatory cytokines, we focused on TNF-α because it has been reported to regulate synaptic plasticity and is implicated in neuropathic pain (Ren et al., 2011; Liu et al., 2017). We observed that TNF-α is upregulated in the mPFC, amygdala, and hippocampus upon CCI. A number of studies have suggested putative roles for brain TNF-α in affective disorders and pain (Dellarole et al., 2014). For instance, aberrant expression of TNF-α in the PFC and hippocampus mediates neuroplastic changes in the brain during neuropathic pain and induces depressive behavior (Kaster et al., 2012). In addition, TNF-α decreases norepinephrine release in the CNS, which inactivates the descending inhibitory pathway facilitating pain transmission (Covey et al., 2000). Such increased TNF-α expression is also known to inhibit BDNF expression and subsequent hippocampal neurogenesis (Fasick et al., 2015; Liu et al., 2017) which may contribute to depressive-like behavioral phenotypes (Bortolato et al., 2015). Of note, the role of spinal cord microglia activation in nerve injury-induced neuropathic pain is sexually dimorphic: nerve injury-induced microglia activation in male mice contributes to central pain sensitization, whereas, in female mice, pain sensitization is independent of spinal microglia activation (Sorge et al., 2015; Mapplebeck et al., 2018). Therefore, it will be interesting to test if there is sexual dimorphism in chronic pain-associated affective disorders and, if so, if brain microglia activation is involved in such sexual dimorphism.

In this study, we detected nerve injury-induced microglial activation in the mPFC, amygdala, and hippocampus in a mouse model. Besides depression, these brain areas are involved in brain functions including cognitive function and anxiety. Of note, it was previously reported that chronic pain often leads to cognitive deficits and anxiety (Gui et al., 2016). Although it is speculative, we hypothesize that brain microglial activation in these brain regions also affects cognitive function such as memory and anxiety.

## Conclusion

In conclusion, our results demonstrate that peripheral nerve injury induces microglial activation and TNF-α expression in the mPFC, amygdala, and hippocampus at delayed time points (4–8 weeks post-injury) after CCI, implicating brain microglial activation in the development of chronic pain-associated depression. Further investigations are needed for an in-depth understanding of the roles and mechanisms of microglia in the development of affective disorders in patients with neuropathic pain.

## Data Availability

All datasets generated for this study are included in the manuscript and/or the supplementary files.

## Author Contributions

W-HC and SL conceived and designed the experiments. EB performed the experiments. W-HC supervised the acquisition of results. EB and W-HC analyzed the data. EB, W-HC, SJ, and SL wrote and refined the article.

## Conflict of Interest Statement

The authors declare that the research was conducted in the absence of any commercial or financial relationships that could be construed as a potential conflict of interest.
